# High CDCP1 Expression Reflects Immune and Stromal Remodeling and Oncogenic Signaling in Pancreatic Ductal Adenocarcinoma

**DOI:** 10.14740/wjon2682

**Published:** 2025-12-17

**Authors:** Kentaro Miyake, Masanori Oshi, Akira Takenouchi, Kota Sahara, Jun Yamamoto, Yutaro Kikuchi, Yu Sawada, Yuki Homma, Ryusei Matsuyama, Itaru Endo

**Affiliations:** a Department of Gastroenterological Surgery, Yokohama City University Graduate School of Medicine, Yokohama, Kanagawa 236-0004, Japan; b These authors contributed equally to this article.

**Keywords:** Biomarker, CDCP1, Genomic instability, Immunosuppression, Pancreatic ductal adenocarcinoma

## Abstract

**Background:**

CUB domain-containing protein 1 (CDCP1) is implicated in pancreatic ductal adenocarcinoma (PDAC) prognosis, but its relationship to the tumor microenvironment (TME) and oncogenic signaling remains incompletely defined. We hypothesized that CDCP1 expression is associated with hallmark cancer signaling pathways and transcriptionally inferred TME remodeling in PDAC.

**Methods:**

We analyzed transcriptomic and clinical data from 214 PDAC cases (The Cancer Genome Atlas (TCGA), n = 145; GSE62452, n = 69). Patients were stratified into high CDCP1 and low CDCP1 groups based on the top tertile of expression. Immune and stromal components of the TME were quantified using the xCell algorithm. Gene Set Enrichment Analysis (GSEA) with Hallmark gene sets was used for pathway enrichment.

**Results:**

High CDCP1 expression was significantly associated with reduced infiltration of CD8^+^ T cells, adipocytes, and fibroblasts, suggesting an immune-excluded and stromally depleted TME. It also correlated with increased homologous recombination deficiency scores, mutation burden, and single-nucleotide variants. CDCP1 expression correlated with CDKN2A mutation but was only weakly associated with KRAS, TP53, and SMAD4 alterations. GSEA showed consistent enrichment of proliferative (E2F, MYC, G2M, p53) and protumorigenic (transforming growth factor-β, hypoxia, glycolysis) pathways in high CDCP1 tumors across both datasets.

**Conclusion:**

CDCP1 defines a transcriptionally distinct PDAC subtype characterized by immune evasion, stromal depletion, and genomic instability. These findings highlight CDCP1 as a potential therapeutic target and biomarker reflecting interplay between oncogenic signaling and the TME.

## Introduction

Pancreatic ductal adenocarcinoma (PDAC) is among the most aggressive and treatment-resistant malignancies, with a 5-year survival rate under 10% [1, 2], and is projected to become the second leading cause of cancer-related deaths in developed regions [2, 3]. Despite improvements in surgical techniques and perioperative management, only a minority of patients are diagnosed at a resectable stage, and even those undergoing potentially curative surgery often relapse early due to micrometastases and therapy resistance. These clinical limitations highlight the need for robust molecular biomarkers that not only reflect tumor-intrinsic aggressiveness but also capture the complex biology of the tumor microenvironment (TME), which in PDAC is notably immunosuppressive and desmoplastic [4]. Such biomarkers could improve prognostic precision and guide the development of targeted therapeutic strategies tailored to both the tumor and its microenvironment.

CUB domain-containing protein 1 (CDCP1) has emerged as a molecule of growing interest in cancer biology, owing to its role in promoting cell adhesion, motility, and survival across multiple epithelial malignancies. As a transmembrane glycoprotein and substrate of Src family kinases, CDCP1 has been implicated in tumor progression and metastasis in colorectal, ovarian, lung, and renal cancers [5-9]. Elevated expression of CDCP1 has been linked to poor prognosis and enhanced invasiveness in several preclinical cancer models. Mechanistically, CDCP1 acts as a substrate of Src family kinases and modulates tyrosin phosphorylation signaling, promoting extracellular matrix remodeling and epithelial-to-mesenchymal transition (EMT) [8]. Through these processes, CDCP1 is thought to influence the composition of the TME, including immune cell and stromal infiltration [4]. However, the transcriptomic correlates of CDCP1 expression in PDAC and its relationship with hallmark oncogenic pathways and TME remodeling remain largely unexplored.

Although CDCP1 has been studied in various cancers, its role in PDAC remains largely unexplored [10-12]. Given the immunosuppressive and stromal-rich microenvironment of PDAC, we hypothesized that CDCP1 expression reflects both tumor-intrinsic features, such as mutational burden and oncogenic pathway activation, and microenvironmental remodeling involving immune and stromal components. Elucidating the molecular and microenvironmental characteristics of high CDCP1 PDAC tumors could provide novel insights into patient stratification and therapeutic vulnerabilities.

The aim of this study was to systematically evaluate the association of CDCP1 expression with TME composition, mutational landscape, and hallmark cancer signaling pathways, and patient survival in PDAC using large-scale transcriptomic datasets. Our findings provide a comprehensive characterization of CDCP1-driven phenotypes and highlight its potential as a prognostic biomarker and therapeutic target.

## Materials and Methods

### Clinical and transcriptomic data of The Cancer Genome Atlas (TCGA) and Gene Expression Omnibus (GEO) cohorts in PDAC patients

We analyzed two publicly available transcriptomic datasets of PDAC patients: The Cancer Genome Atlas Pancreatic Adenocarcinoma (TCGA-PAAD, n = 145) and the GSE62452 cohort (n = 69). The mRNA expression profiles and clinical data for the TCGA cohort were obtained from the cBioPortal and the Pan-Cancer Clinical Data Resource. The GSE62452 dataset was downloaded from the GEO database. All gene expression data were log2-transformed prior to analysis.

### Pathway, TME, and genomic alteration analysis

Patients were stratified into high CDCP1 and low CDCP1 groups based on tertile distribution within each cohort, with the top tertile defined as high and the remaining two tertiles combined as low. To investigate pathway activity, we conducted Gene Set Enrichment Analysis (GSEA) using the Hallmark gene sets from the Molecular Signatures Database (MSigDB v7.5.1) [13]. Pathways with a normalized enrichment scores (NES) > 1.5 and false discovery rates (FDR) < 0.25 were considered significantly enriched. In addition, Gene Set Variation Analysis (GSVA) was performed using the GSVA package in R to calculate enrichment scores for selected hallmark pathways, including E2F targets, G2M checkpoint, MYC targets, mitotic spindle, mTORC1 signaling, p53 pathway, glycolysis, and hypoxia.

To estimate TME composition, the xCell algorithm was applied to bulk transcriptome data to infer the relative abundance of 64 immune and stromal cell types [14].

For the TCGA cohort, we further examined genomic instability features [15]. Mutation data were used to calculate silent and non-silent mutation rates, single nucleotide variant (SNV) burden, and insertion/deletion (indel) neoantigen loads. Homologous recombination deficiency (HRD) scores, intratumor heterogeneity scores, and fraction genome altered were obtained from previously published pan-cancer studies [16-19]. We assessed the association between CDCP1 expression and mutation status of canonical PDAC driver genes (KRAS, TP53, CDKN2A, and SMAD4).

### Statistical analysis

R software (version 4.0.1, R Project for Statistical Computing) and Microsoft Excel (version 16 for Windows, Redmond, WA, USA) were utilized to perform data analysis and generate graphical representations for this study. Comparisons between groups were evaluated using Kruskal-Wallis test or the Mann-Whitney U test, as appropriate. Categorical variables were assessed using Fisher’s exact test. Survival analysis was carried out using the Log-rank test. A P-value of less than 0.05 was considered statistically significant.

This study was conducted entirely *in silico* and did not involve human or animal subjects.

## Results

### High CDCP1 expression predicts poor prognosis in PDAC

**Figure 1:**
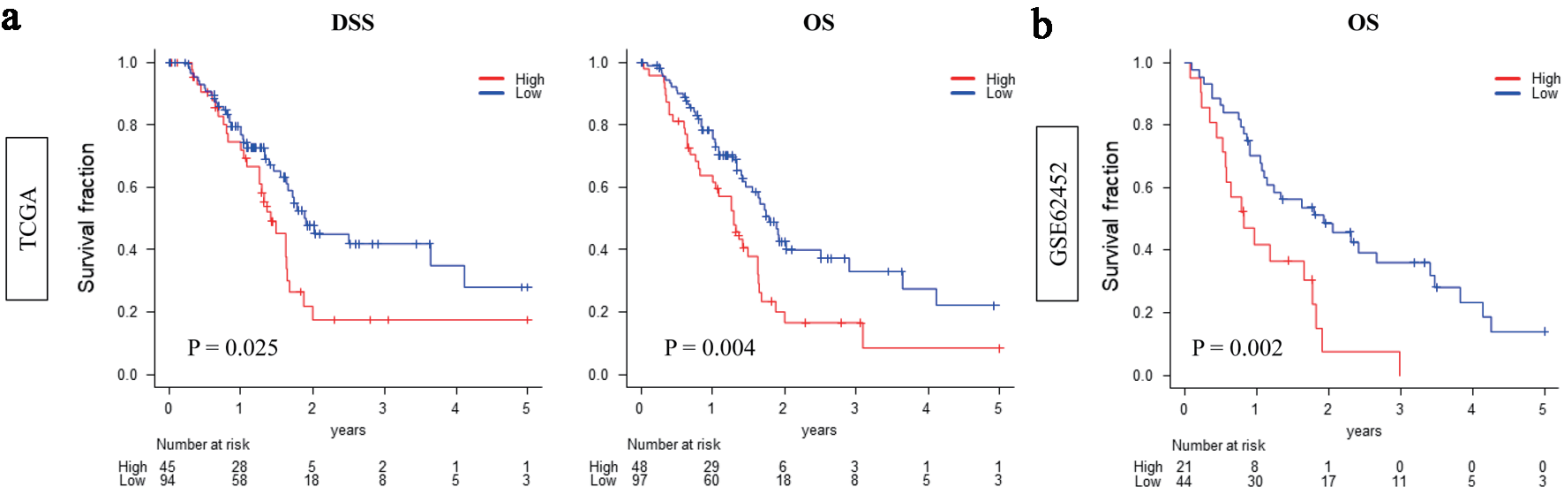
High CDCP1 expression is associated with poor survival in pancreatic ductal adenocarcinoma (PDAC). Kaplan-Meier survival curves comparing overall survival between high CDCP1 and low CDCP1 groups in the TCGA and GSE62452 cohorts. Survival endpoints include disease-specific survival (DSS) and overall survival (OS) in TCGA (a) and OS in GSE62452 (b). Log-rank test was used to evaluate statistical significance.

Kaplan-Meier analysis demonstrated that patients with high CDCP1 expression had significantly worse overall survival compared to those with low CDCP1 expression, both in the TCGA cohort (median: 14.2 vs. 22.7 months; P = 0.004) and the GSE62452 cohort (median: 12.6 vs. 20.5 months; P = 0.002) (Fig. 1).

### High CDCP1 PDAC shows decreased infiltration of cytotoxic T cells

**Figure 2:**
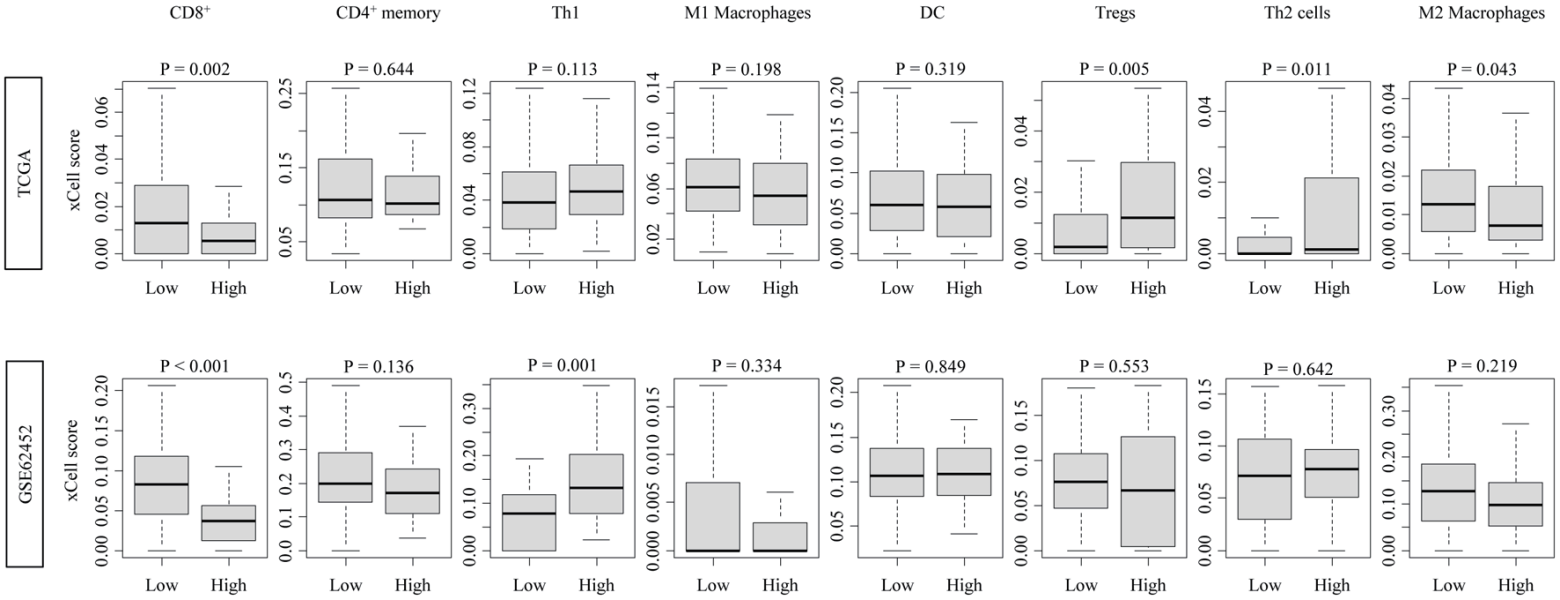
Immune cell infiltration profiles associated with CDCP1 expression in PDAC. Boxplots showing xCell-inferred infiltration scores of CD8^+^ T cells, CD4^+^ memory T cells, Th1 cells, Th2 cells, regulatory T cells (Tregs), dendritic cells (DCs), and M1/M2 macrophages in high CDCP1 and low CDCP1 groups in the TCGA and GSE62452 cohorts.

High CDCP1 PDAC tumors exhibited significantly reduced infiltration of CD8^+^ T cells, consistently in both cohorts (TCGA: P = 0.002; GSE62452: P < 0.001). In contrast, other lymphocyte populations, including regulatory T cells (Tregs), CD4^+^ T cells, and natural killer (NK) cells, showed minimal or no association with CDCP1 expression. These findings suggest a specific impairment of cytotoxic immune infiltration in high CDCP1 PDAC (Fig. 2).

### High CDCP1 PDAC shows reduced infiltration of stromal cells

**Figure 3:**
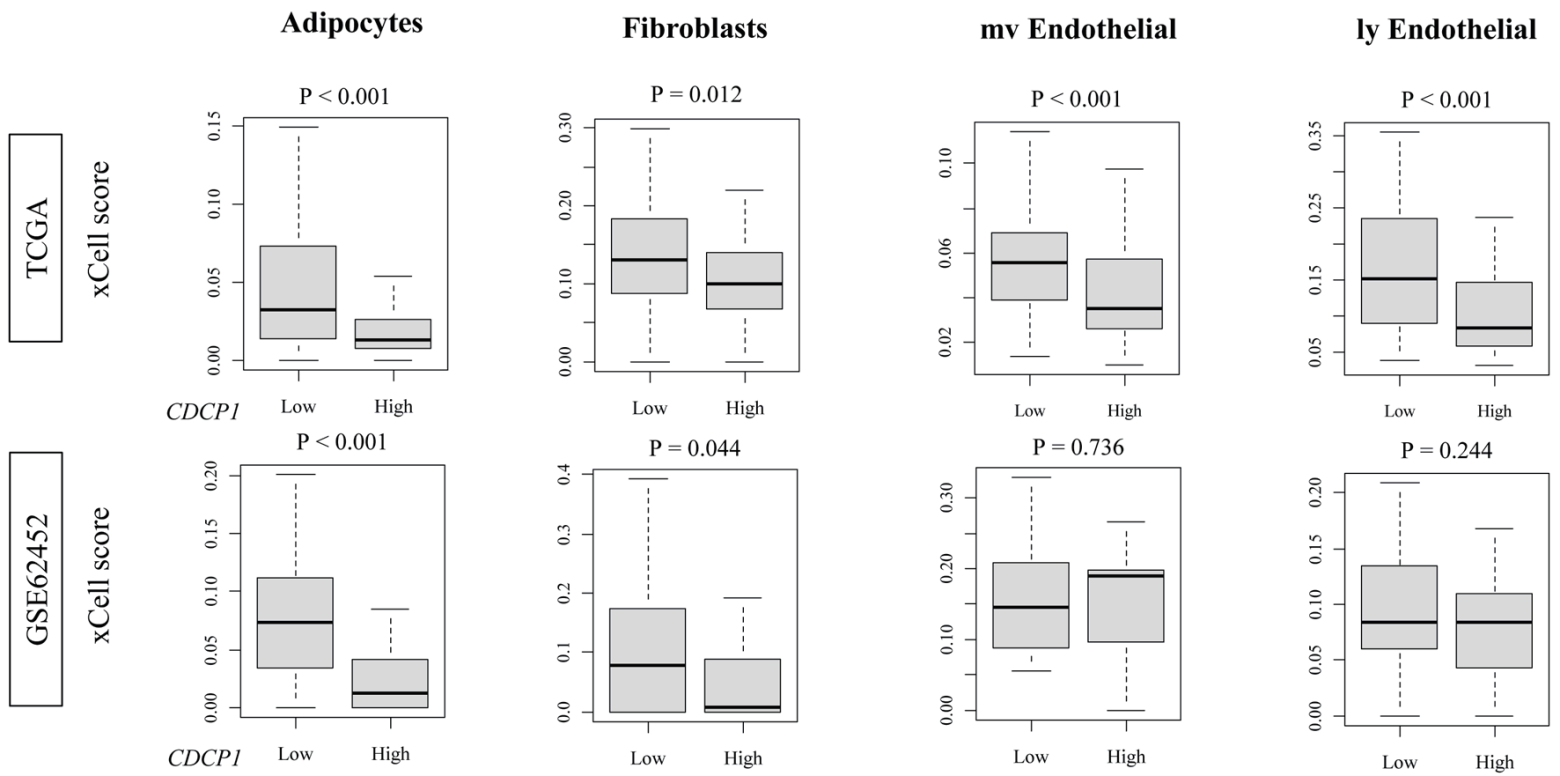
Stromal and endothelial cell fractions in high CDCP1 versus low CDCP1 PDAC tumors. xCell scores comparing infiltration of adipocytes, fibroblasts, microvascular endothelial cells (mvECs), and lymphatic endothelial cells (lyECs) between high CDCP1 and low CDCP1 tumors in the TCGA and GSE62452 cohorts.

High CDCP1 PDAC exhibited significantly decreased infiltration of adipocytes (TCGA and GSE62452: both P < 0.001) and fibroblasts (TCGA: P = 0.012; GSE62452: P = 0.044), as estimated by xCell. In contrast, no significant differences were observed in the abundance of endothelial cells or myofibroblasts (Fig. 3). These results suggest a selective depletion of stromal components, particularly lipid-rich and matrix-producing cell types, in the high CDCP1 PDAC TME.

### High CDCP1 PDAC is significantly enriched for protumorigenic pathways

**Figure 4:**
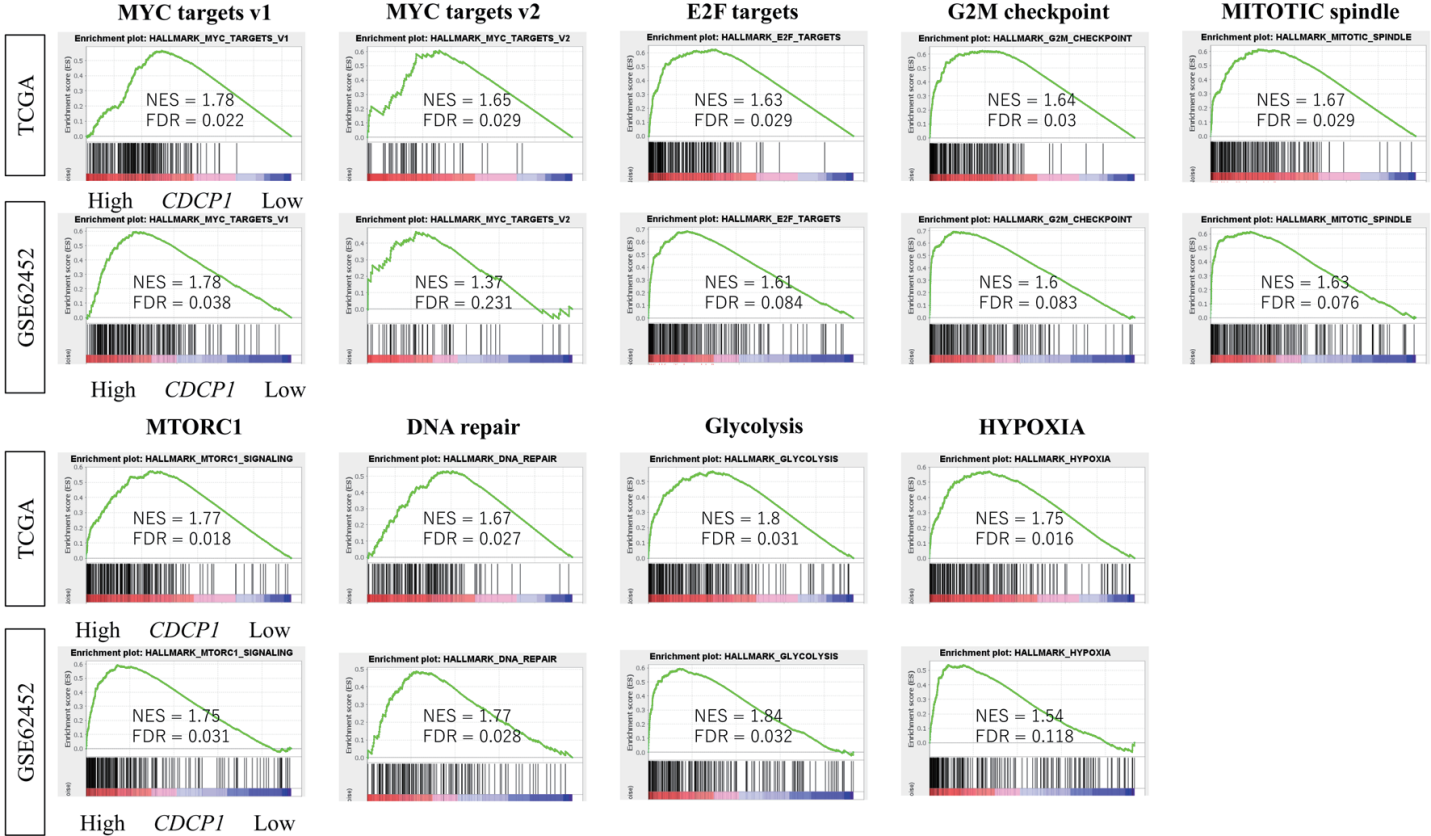
Enrichment of oncogenic signaling pathways in high CDCP1 PDAC. Comparison of Gene Set Variation Analysis (GSVA) scores for hallmark pathways including MYC targets v1/v2, E2F targets, mitotic spindle, G2M checkpoint, mTORC1, DNA repair, PI3K/AKT/mTOR signaling, glycolysis, and hypoxia, between high CDCP1 and low CDCP1 tumors in the TCGA and GSE62452 cohorts.

GSEA revealed that high CDCP1 tumors were significantly enriched for cell proliferation-related gene sets (E2F targets, G2M checkpoint, MYC targets v1/v2, and p53 pathway) as well as protumorigenic processes such as hypoxia, transforming growth factor-β (TGF-β) signaling, and glycolysis. These enrichments were consistently observed in both the TCGA and GSE62452 cohorts (FDR < 0.25; NES > 1.5) (Fig. 4).

### CDCP1 expression in PDAC tumors correlates with genomic instability

**Figure 5:**
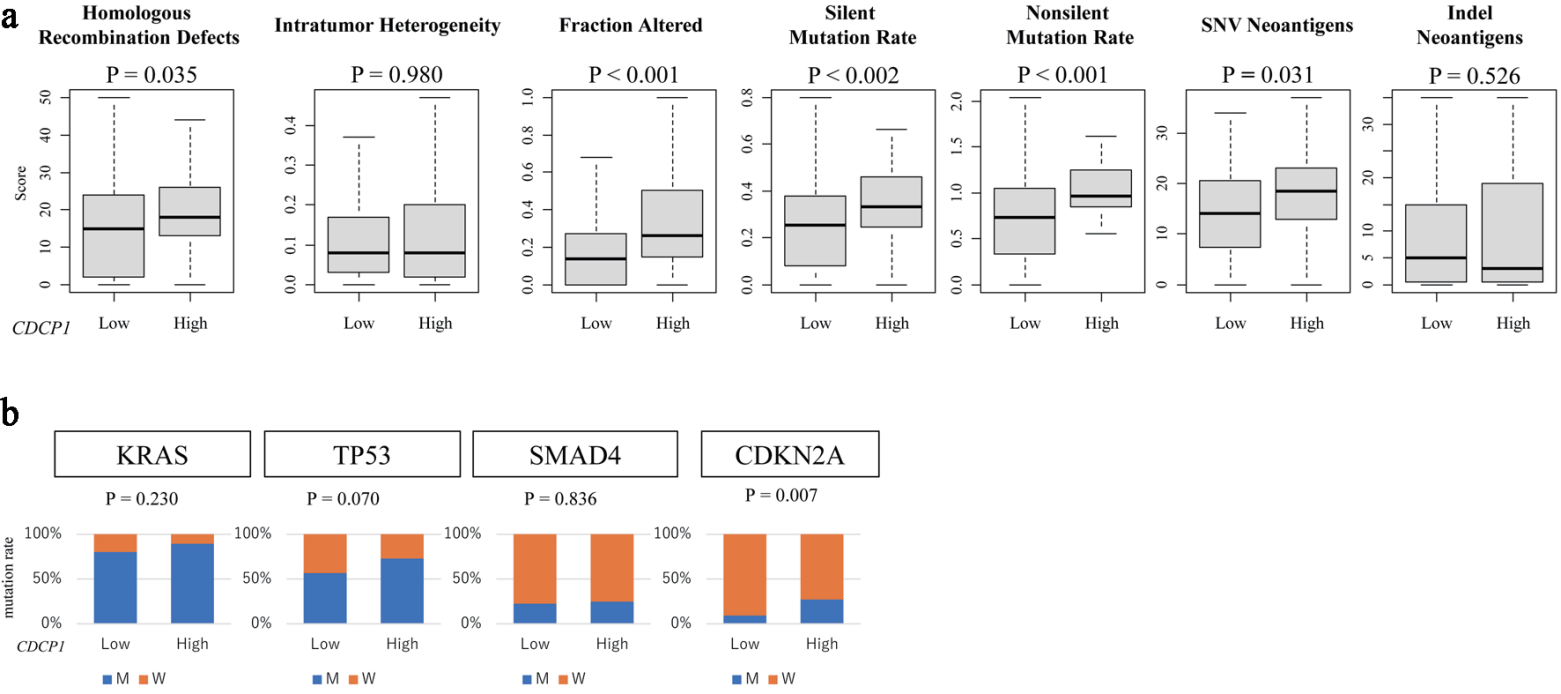
CDCP1 expression is associated with genomic instability and specific gene mutations. (a) Genomic instability metrics including silent and non-silent mutation rates, single nucleotide variant (SNV) and indel neoantigen loads, fraction altered, intratumor heterogeneity, and homologous recombination deficiency (HRD) scores across CDCP1 expression groups. (b) Bar plots of mutation frequency for key PDAC driver genes (KRAS, TP53, SMAD4, and CDKN2A) in high CDCP1 and low CDCP1 tumors in the TCGA cohort.

In the TCGA cohort, high CDCP1 tumors were characterized by increased genomic instability, reflected in significantly higher HRD scores (P = 0.035), elevated silent (P = 0.002) and non-silent (P < 0.001) mutation rates, and SNV burden (P = 0.031). These findings suggest that high CDCP1 PDAC is associated with a hypermutated and genomically unstable tumor phenotype (Fig. 5a).

### Association of CDCP1 with specific gene mutations

Among key driver mutations in PDAC, CDCP1 expression was significantly associated with CDKN2A mutation status (P = 0.007), whereas no such association was observed for KRAS (P = 0.18), TP53 (P = 0.09), or SMAD4 (P = 0.22). This selective correlation indicates a potential link between CDCP1 and dysregulation of cell cycle regulatory pathways, particularly involving the CDKN2A-p16INK4a axis (Fig. 5b).

### Summary of integrated findings

Taken together, high CDCP1 expression in PDAC defines a distinct molecular phenotype characterized by a combination of immunosuppressive TME, reduced stromal composition, enhanced genomic instability, and activation of multiple oncogenic signaling pathways, collectively shaping an aggressive tumor state associated with poor clinical outcome.

## Discussion

This study revealed that high CDCP1 expression in PDAC is linked to poor prognosis and is associated with key biological hallmarks, including reduced cytotoxic immune infiltration, stromal depletion, genomic instability, and activation of multiple protumorigenic signaling pathways. These findings suggest that CDCP1 plays a multifaceted role in shaping both tumor-intrinsic features and the TME in PDAC.

CDCP1 has previously been implicated in tumor progression and metastasis across multiple malignancies, including colorectal, lung, and ovarian cancers [20-22]. However, its clinical and biological significance in PDAC has remained unclear. Our findings extend previous knowledge by demonstrating a link between CDCP1 expression and specific aspects of the PDAC TME, particularly the suppression of cytotoxic immune infiltration and reduction of stromal components such as fibroblasts and adipocytes [4, 23].

Given the established importance of the TME in PDAC therapy resistance and immune evasion [4, 14], our results suggest that CDCP1 is not merely a marker, but may act as a driver of PDAC microenvironmental remodeling. These findings are consistent with previous reports highlighting the critical impact of stromal and immune alterations on PDAC progression and treatment response [4, 23].

The observed association between high CDCP1 expression and HRD is particularly noteworthy. HRD, a hallmark of defective DNA repair, has been linked to increased sensitivity to poly-ADP-ribose polymerase (PARP) inhibitors [24]. While BRCA1/2 mutations are prototypical causes of HRD, our findings suggest that CDCP1 expression could represent a non-BRCA indicator of genomic instability in PDAC [24-26]. Notably, the specific association with CDKN2A mutations, but not with KRAS or TP53, points to a potential involvement of CDCP1 in dysregulated cell cycle control and senescence pathway [27, 28]. Interestingly, among commonly mutated PDAC genes, only CDKN2A mutation showed a significant association with high CDCP1 expression. CDKN2A encodes p16^INK4a^, a tumor suppressor that negatively regulates the G1/S cell cycle checkpoint. Its loss may facilitate uncontrolled proliferation, particularly in the context of CDCP1-driven oncogenic signaling. The co-occurrence of CDCP1 overexpression and CDKN2A mutation may thus define a PDAC subset with heightened proliferative capacity and an immune-excluded phenotype.

Enrichment of multiple hallmark oncogenic pathways, including E2F targets, G2M checkpoint, MYC targets, p53 signaling, hypoxia, TGF-β signaling, and glycolysis [29, 30], reinforces the concept that high CDCP1 tumors possess an aggressive molecular phenotype characterized by enhanced proliferation and metabolic reprogramming [31, 32]. Importantly, GSEA was consistent with enrichment of hypoxia and TGF-β signaling pathways in CDCP1-high tumors. These pathways are known to contribute to immune exclusion and desmoplasia in PDAC, raising the hypothesis that CDCP1 may modulate multiple aspects of tumor progression [31-33]. Based on transcriptomic features such as enrichment of cell cycle-related pathways, reduced cytotoxic immune infiltration, and stromal depletion, CDCP1-high PDAC tumors appear to align with the “basal-like” or “quasi-mesenchymal (QM)” molecular subtypes described by Collisson et al [34]. These subtypes are associated with aggressive tumor behavior, immune exclusion, and limited response to standard therapies. The overlap between CDCP1-driven phenotypes and the basal-like subtype supports the potential utility of CDCP1 as a surrogate marker for molecular classification and may inform therapeutic stratification strategies in PDAC.

Clinically, CDCP1 may function as both a prognostic biomarker and a therapeutic target. CDCP1-targeting antibody-drug conjugates (ADCs) have shown promising preclinical efficacy in PDAC models [8], and patients with high CDCP1 expression may derive particular benefit. CDCP1 is a single-pass transmembrane glycoprotein functioning as a central signaling hub promoting tumor proliferation, invasion, and metastasis. It interacts with receptor tyrosine kinases such as epidermal growth factor receptor (EGFR), integrins, downstream effectors including Src, PKCδ, Akt, and FAK [12]. In PDAC, CDCP1 has been linked to the basal-like molecular subtype, which is associated with poor prognosis and aggressive clinical behavior. Furthermore, CDCP1 facilitates EMT and induces the expression of matrix metalloproteinases such as MMP-9, promoting basement membrane degradation and stromal remodeling [12].

In preclinical studies, CDCP1-targeted therapies, including ADCs, radiolabeled antibodies, and bispecific T-cell engagers, have suppressed tumor growth in PDAC and other solid tumors [7, 8, 35]. In particular, antibodies specific for cleaved CDCP1 (c-CDCP1), a neoepitope specific to tumor cells, have shown high specificity and minimal off-target toxicity. Given the observed association between CDCP1 expression and HRD, combining CDCP1-targeted therapies with DNA-damaging agents or PARP inhibitors may warrant further investigation [24].

Our study has several limitations. First, it is based on retrospective analyses of publicly available datasets [14, 15, 36]. Second, TME composition was inferred computationally and not confirmed by histopathological or flow cytometric analyses [14]. While this limitation is acknowledged, we have now further emphasized the need for future validation using histological or single-cell techniques to confirm our computational inferences. Third, although we identified associations between CDCP1 expression and several oncogenic features, causality cannot be established from transcriptomic data alone.

In addition, a key limitation of this study is the absence of cross-validation using an independent PDAC cohort such as International Cancer Genome Consortium (ICGC) or other GEO datasets. Although our findings were consistent across two well-established cohorts (TCGA and GSE62452), further validation in additional datasets with sufficient transcriptomic and TME information is warranted to strengthen the generalizability of our results.

Future mechanistic studies are warranted to elucidate how CDCP1 modulates immune exclusion, stromal remodeling, and genomic instability in PDAC.

In conclusion, CDCP1 expression is associated with immune evasion, stromal depletion, genomic instability, and activation of pro-cancerous pathways in PDAC. CDCP1 thus emerges as a promising biomarker and therapeutic target, providing a basis for further translational and clinical investigations.

### Conclusion

In summary, high CDCP1 expression in PDAC is associated with poor patient survival, reduced CD8^+^ T-cell infiltration, decreased stromal cellular fractions, increased genomic instability, and activation of multiple oncogenic signaling pathways. These findings are consistent with that CDCP1 plays a crucial role in both tumor-intrinsic aggressiveness and the modulation of the TME. CDCP1 may serve as a novel prognostic biomarker and potential therapeutic target in PDAC. Future studies are warranted to validate these findings and to explore the clinical utility of CDCP1-directed therapeutic strategies.

## Data Availability

The data supporting the findings of this study are available from the corresponding author upon reasonable request.
